# Quantitative Network Measures as Biomarkers for Classifying Prostate Cancer Disease States: A Systems Approach to Diagnostic Biomarkers

**DOI:** 10.1371/journal.pone.0077602

**Published:** 2013-11-13

**Authors:** Matthias Dehmer, Laurin A. J. Mueller, Frank Emmert-Streib

**Affiliations:** 1 UMIT, Institute for Bioinformatics and Translational Research, Hall in Tyrol, Austria; 2 Computational Biology and Machine Learning Laboratory, Center for Cancer Research and Cell Biology, School of Medicine, Dentistry and Biomedical Sciences, Queen's University Belfast, Belfast, United Kingdom; University of Catania, Italy

## Abstract

Identifying diagnostic biomarkers based on genomic features for an accurate disease classification is a problem of great importance for both, basic medical research and clinical practice. In this paper, we introduce quantitative network measures as *structural biomarkers* and investigate their ability for classifying disease states inferred from gene expression data from prostate cancer. We demonstrate the utility of our approach by using eigenvalue and entropy-based graph invariants and compare the results with a conventional biomarker analysis of the underlying gene expression data.

## Introduction

Molecular and clinal biomarkers have been investigated extensively in medicine and related areas [1], [2], [3], [4]. In particular, biomarkers have been used for cancer analysis, cancer screening and stratification and diagnosis [1], [2], [3], [4]. Classically, diagnostic biomarkers represent molecules such that their occurrence or concentration in tissue samples or blood is representative for a certain cancer state, see [5]. Numerous studies have been performed for demonstrating the usefulness and impact of such biomarkers in cancer research and related fields [1], [2], [3], [4].

The above mentioned results dealing with biomarker research are based on the widely accepted classical view that differentially expressed genes can be interpreted as markers of diseases. However, recent research revealed that classical single-gene biomarker are often less meaningful for analyzing diseases than using network-based biomarker, see [6], [7], [8], [9]. Here, pathways representing complex networks [10], [6], [7] serve as biomarkers of diseases. We now briefly sketch relevant related work of so-called network-based biomarkers as follows. For instance, a protein-network-based method for identifying biomarkers subnetworks inferred from protein interaction databases has been developed by Chuang et al. [11]. This method has been proven useful when classifying these subnetworks for disease signature discrimination [11]. A similar approach due to Chen et al. [12] to prioritize disease genes and protein interaction subnetworks turned out to be useful too as these subnetworks can discriminate disease signatures. Guyon et al. [8] used support vector machine classification such that the method takes network interactions into account rather than only single genes. Jin et al. [9] interpreted certain subgraphs, for example triangle graphs, as protein biomarkers and performed a statistical analysis thereof, see [9]. Finally Barabási et al. [13] used, e.g., structural properties of graphs by using centrality measures and degree distributions to find network-based biomarkers via feature selection.

In this paper, we introduce quantitative network measures as structural biomarkers and investigate their ability when classifying disease states inferred from prostate cancer (see section ‘Data’). The problem of finding appropriate network measures which capture structural information uniquely and, therefore, the problem of identifying suitable candidates as structural biomarkers is intricate. This relates to the open problem that it is not a priori clear what kind of structural features could be best as there are infinitely many features that are graph invariants [14], [15] to characterize the structure of pathways (complex networks), see also [14], [16], [17], [18].

The major contribution of this paper is as follows. We use eigenvalues of biological networks inferred from prostate cancer microarray data as structural biomarkers by using supervised learning. More precisely, we demonstrate that these structural biomarkers, representing eigenvalue-based graph invariants, can be used to classify prostate cancer meaningfully; in this context we obtain reasonable results when classifying cancer vs. benign tissue, see also [19].

## Methods

### Structural Biomarkers

In this paper, we introduce quantitative network measures as structural biomarkers. That means by starting from biological networks inferred from microarray data (see section ‘Data’), we calculate quantitative graph measures representing network complexity measures and employ supervised learning. If these structural features can classify/discriminate disease states, they are referred to as structural biomarkers. In fact, this opens new perspectives in biomarker research as (i) infinitely many structural features (e.g., graph invariants) exist for structural network characterization and (ii) there exist several machine learning and statistical methods to use the derived structural features for classification/discrimination.

As structural biomarkers, we are going to use eigenvalue- and entropy-based quantities. We start by explaining the procedure to derive eigenvalue-based graph invariants. If 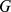 denotes a network, then eigenvalue-based measures can be calculated by using a graph-theoretical matrix 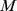 [20] inferred from 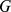. Finally we yield.
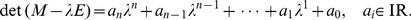
(1)


In this paper, we set 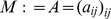 and 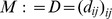. 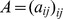 is the adjacency matrix and 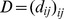 is the distance matrix, respectively [17], [20]. By solving the algebraic equation.
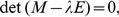
(2)we obtain the non-zero eigenvalues 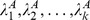 and 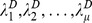. As 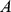 and 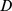 are symmetrical for undirected graphs, it holds 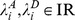. From the sketched calculation of the eigenvalues by using 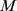 inferred from 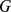, we define the measures [17], [21], [22]:



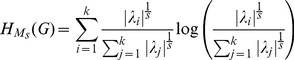
(3)

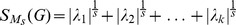
(4)

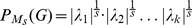
(5)

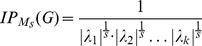
(6)

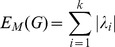
(7)and



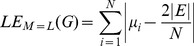
(8)

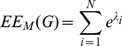
(9)

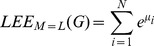
(10)

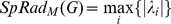
(11)


In order to calculate the measures concretely by using R, we set 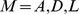. 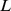 is the laplacian of 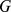 and 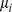 are its eigenvalues thereof [23].

The second class of graph measures we employ as structural biomarkers represent entropy measures for graphs. These measures have been investigated extensively by Dehmer et al. [24], [25], [26] and originally by Mowshowitz [27], [28], [29], [30]. Such measures rely on Shannon's entropy and, hence, a probability distributions must be assigned to a graph 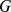. This problem is intricate as, again, infinitely many structural features exist (e.g., vertex degrees, vertices, edges, distances, and partitions thereof) to define entropic measures on a network.

Basically, two methods exist to infer a probability distribution of a graph by taking its structural features into account. The first method is based on determining partitions by using an arbitrary graph invariant and equivalence criterion [31], [27]. The second procedure is based on using so-called information functionals and on assigning a probability value to every vertex. Properties of graph entropies based on both methods have been investigated in [24], [25], [26], [16]. As a result of the extensive research in this field of the last three decades, numerous graph entropy measures have been developed, see, e.g., [31], [32], [24], [27], [33], [34]. It would go beyond the scope of the paper to examine all existing graph entropy measures as candidates for structural biomarker. Nevertheless, we used the following entropies from different paradigms (as a result of the feature selection process, see also section ‘Results’) [31], [24]:

1. Dehmer entropy by using the information functional 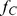 (vertex centrality), see [24].
2. Topological Information Content [35].
3. Graph Vertex Complexity Index [36].
4. Mean information content of distance-degree equality [31].
5. Mean information content on the edge equality [31].
6. Balaban index 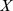 [37].
7. Entropic symmetry index [38].
8. Bonchev index 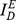 [31].
9. Dehmer-entropy by using the information functional 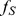 (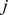-spheres), see [24].
10. Bonchev index 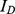 [31].

The concrete formulas thereof and the technical details can be found in [31], [24].

### Data

The data set we use in this paper has never been used for classification cancer disease states. To create the set of biological networks, we used seven publicly available data sets (see Table 1) related to prostate cancer from NCBI GEO [39] and EBI Arrayexpress [40]. The data sets have been selected in cooperation with the Urology Department at the Medical University Innsbruck to identify transcriptional changes in prostate cancer, including tumors with ERG gene rearrangements, see [19]. A first result by using this data has been achieved by Massoner et al. [19] as they found robust population-independent transcriptional changes and signs of ERG rearrangements inducing metabolic changes in cancer cells by activating major metabolic signaling molecules like NPY.

**Table 1:** This table lists the public data sets we used to infer this set of biological networks.

			Number of Samples	
Author	Ref	Platform	Benign	Cancer
Chandran et al.	[50]	Affymetrix HG-U95av2	11	50
Liu et al.	[51]	Affymetrix HG-U133a	13	44
Sing et al.	[52]	Affymetrix HG-U95av2	37	48
Tsavachidou et al.	[53]	Affymetrix HG-U133a	40	16
Wallace et al	[54]	Affymetrix HG-U133a2	14	53
Varambally et al.	[55]	Affymetrix HG-U133 2+	4	6
Yu et al.	[56]	Affymetrix HG-U95av2	56	51

We reanalyzed the publicly available data sets (see Table 1) and inferred biological networks by using the C3NET inference method [41]. This resulted in seven C3NET networks 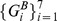 representing the benign tissue (from the control group) and seven networks 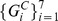 representing cancer tissue. Here, benign means that we refer to sick patients with a tumor.

In order to obtain a larger set of networks, we used the gene ontology (GO) database [42] to extract subgraphs from these 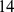 networks. For each network and each GO-term in the category ‘biological process’, we extract one subgraph containing the genes associated with this specific GO-term resulting in 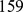 and 108 networks representing benign and cancer tissue, respectively. We determined the GO-terms by using the Bioconductor Package goProfiles.

The resulting sizes of the obtained classes are potentially different because the network structures of 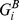 and 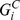 are different and, hence, not all pathways are captured by these networks. Furthermore, we exclude a subnetwork whenever it contains less that 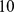 genes associated with a specific GO-term. The obtained two sets of networks can be interpreted as an approximation of two populations. One population represents the *benign* state and the second the *cancerous* state. We note that this set of biological networks has already been used in [43] when demonstrating the functionality of the recently developed R-package QuACN.

## Results

### Classification: Prostate Cancer Networks vs. Gene Expression Biomarkers

In order to evaluate the performance of the new structural biomarkers, we compare the classification of the networks with the classification of the gene expression data itself by using supervised learning. To classify the normalized gene expression data by using the data sets described in section ‘Data’, we combined the samples of the seven studies (see Table 1) by determining the intersection of the measured genes. This results in a feature vector that contains all genes that are measured in each of the seven different studies. In order to select the most important genes, we apply a feature selection mechanism based on the *information gain* method [44]. Then we classify the data set by using the 10 most important features as a feature vector by using SVM classification [45] with a polynomial kernel function. For performing the classification, we apply the R-implementation of Libsvm [46] and for learning the optimal parameters, we perform a 10-fold cross validation.

In order to obtain the best classification performance we assess the following parameter settings for the classification exhaustively:
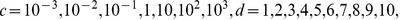
(12)and



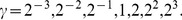
(13)For the three studied measures, their results in form of error measures of the classification are summarized in Table 2. For these measure, we found the optimal parameter settings used for this analysis: 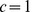, 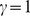, 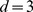 (eigenvalue-based measures), 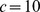, 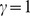, 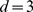 (entropy-based measures) and 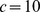, 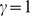, 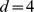 (gene expression data).

**Table 2:** Error measures (mean values and standard errors) for the evaluation of the classification. Best results are highlighted in bold.

	Eigenvalue		Entropy		Biomarker	
	mean	sd error	mean	sd error	mean	sd error
Sensitivity	**0.79**	0.04	0.71	0.01	**0.83**	0.03
Specificity	**0.79**	0.04	0.71	0.01	**0.83**	0.03
Precision	**0.72**	0.16	0.68	0.12	**0.81**	0.07
Recall	**0.79**	0.04	0.71	0.01	**0.83**	0.03
Accuracy	0.76	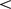 0.01	0.71	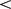 0.01	**0.85**	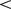 0.01
F-Score	0.72	0.07	0.68	0.07	**0.82**	0.05

From our numerical classification of the data, summarized in Table 2, it follows that the network approach based on eigenvalues (second column) and the biomarker analysis of the gene expression data (forth column) perform best. Specifically, the classification of the gene expression biomarkers is always best but the eigenvalue method results in a comparable performance, within one standard error. Due to the fact that all error measures are random variables, estimated from a 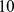-fold cross validation, it appears sensible to consider *performance intervals*, given by the mean and standard error, rather than point estimators. This will lead to more robust statements regarding the obtained performance values.

In contrast to the eigenvalue and gene expression biomarker method, the classification method based on the entropies of networks results in the lowest performance for all error measures, however, still giving a sensible classification performance indicating that also this method is capable for discriminating the two biological classes, at least to a certain extent.

### Eigenvalue-based Structural Analysis of the Prostate Cancer Networks

**Figure 1:**
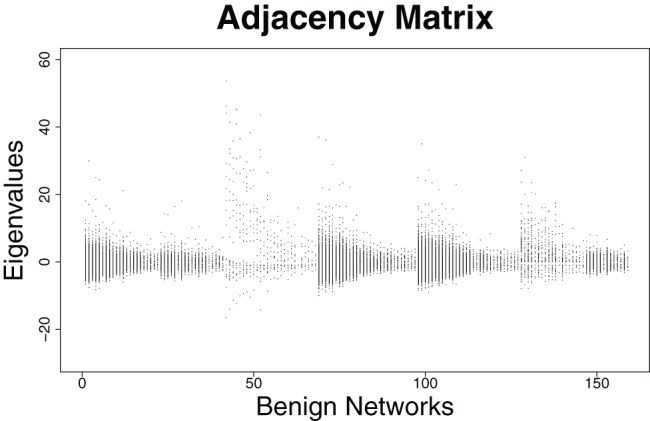
Distributions of the Eigenvalues of the adjacency matrix for the *benign* networks.

**Figure 2:**
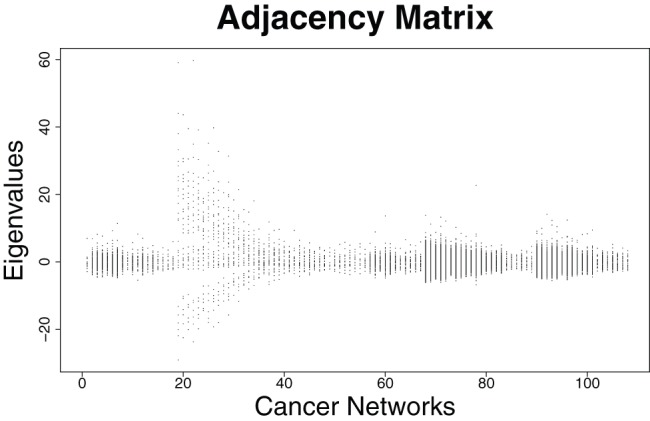
Distributions of the Eigenvalues of the adjacency matrix for the *cancer* networks.

**Figure 3:**
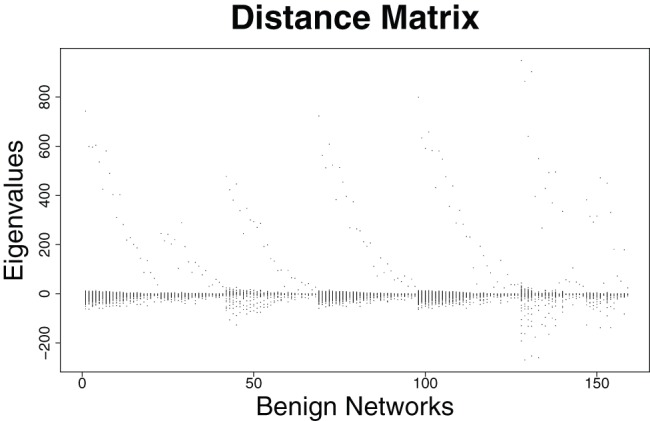
Distributions of the Eigenvalues of the distance matrix for the *benign* networks.

In this section, we examine some properties of the eigenvalues by using the prostate cancer networks representing two classes (cancer and benign tissue). First results are summarized in Figure 1, 2 and Figure 3, 4. We plotted all eigenvalues for the cancer and benign networks by employing the adjacency and distance matrix, respectively. By using the adjacency matrix, the eigenvalues of the benign networks show a characteristic distribution where nearly all eigenvalues are situated in a horizontal strip. In fact, 64% of these eigenvalues are negative and 36% are positive. The plot of the cancer networks by employing the adjacency looks very similar. Here, the ratio of positive and negative eigenvalue is the same as by using the benign networks. The fact that these distributions look similar can be also explained by arguing with the corresponding zero-free regions (e.g., strip-like regions in which no zeros of the characteristic polynomial lie). As mentioned in section ‘Structural Biomarkers’, eigenvalues are the zeros (that means the solutions of the equation 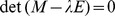) of the characteristic polynomial by using a graph-theoretical matrix 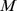 (here, we use 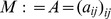 and 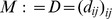). Then, we see that the zero-free regions of benign vs. cancer networks by using the adjacency matrix look very similar. But from this, we cannot conclude that eigenvalues are generally unsuitable for discriminating the two network classes as seen in section ‘Classification: Prostate Cancer Networks vs. Gene Expression Biomarkers’. By using the distance matrix, we yield the eigenvalue-ratios 74% negative and 26% positive for benign; 76% negative and 24% for cancer networks. In contrast to the distributions by using the adjacency matrix, the horizontal strips and, hence, the zero-free regions are different. This can be understood by analyzing the distributions of the matrix elements of the adjacency and distance matrix. The fact that those are different also implies that the coefficients of the resulting characteristic polynomials differ significantly.

**Figure 4:**
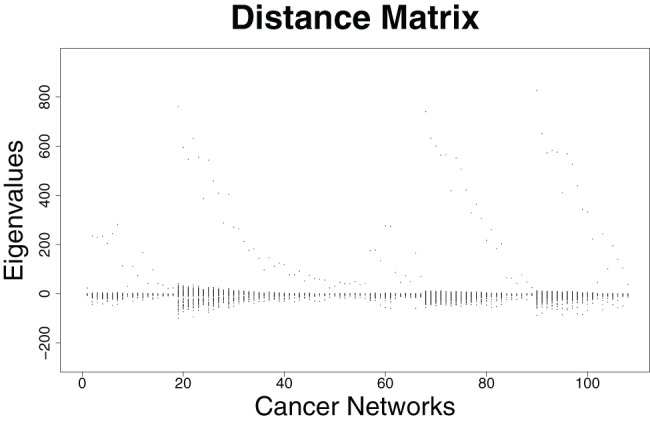
Distributions of the Eigenvalues of the distance matrix for the *cancer* networks.

In summary, we may conclude that certain eigenvalue-based measures by using the adjacency and distance matrix capture structural information differently. Here, this could mean that some of these measures by using the distance matrix are more sensitive toward slight structural changes in the network. The validity of this hypothesis can be underpinned by evaluating the discrimination power of eigenvalue-based measures. This relates to determine whether the measure captures structural information uniquely, see [47], [16], [14]. For instance, if the network structure is slightly altered, the measure should detect this structural change by giving distinguishable values. In this paper, we measure the discrimination power or uniqueness by the quantity, 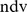, expressing the *non-distinguishable values* by a particular eigenvalue-based measure. That is to calculate ndv, we compute all measures on the networks and determine the number of graphs which cannot be distinguished by them. Importantly, the networks need to be structurally non-equivalent (non-isomorphic) to perform this study meaningfully; we emphasize that the cancer networks used in this study have been checked to be structurally non-equivalent. By inspecting Table 3, we see first of all that many of the computed eigenvalue-based measures are fully unique; *to normalize the values, we employed Konstantinova's sensitivity measure 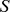, see [48], [17].* That means they structurally distinguish the networks by their values uniquely. The only measure that produces degenerate values is 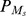, see Equation 5. Moreover, we observe that 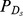 is more unique than 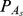 that can be seen by the ndv-values. Thus, we may conclude that the distance matrix encodes structural information more meaningfully than by using the adjacency matrix when employing the measure 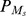.

**Table 3:** ndV-values for the structural biomarkers (eigenvalue and entropy-based measures) for all prostate cancer networks and the corresponding subgroups (benign/cancer).

	absolute			relative		
	all	benign	cancer	all	benign	cancer
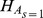	0	0	0	0	0	0
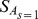	0	0	0	0	0	0
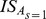	0	0	0	0	0	0
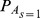	141	80	58	0.53	0.50	0.54
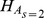	0	0	0	0	0	0
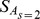	0	0	0	0	0	0
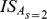	0	0	0	0	0	0
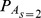	115	66	46	0.43	0.42	0.43
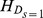	0	0	0	0	0	0
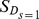	0	0	0	0	0	0
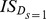	0	0	0	0	0	0
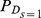	15	8	7	0.06	0.05	0.06
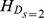	0	0	0	0	0	0
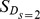	0	0	0	0	0	0
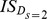	0	0	0	0	0	0
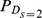	9	4	3	0.03	0.03	0.03
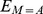	0	0	0	0	0	0
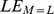	0	0	0	0	0	0
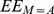	0	0	0	0	0	0
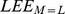	0	0	0	0	0	0
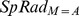	0	0	0	0	0	0

Note that the supplementary files (File S1, S2, S3) contain the values of the calucated networks.

## Discussion and Conclusion

Within recent years there is a considerable interest in the identification of biomarkers within genomic datasets. Usually, if gene expression data are used from DNA microarray experiments, a biomarker is considered as a gene, or a set of genes, for which gene expression data are available. Then, classification methods are based on the gene expression data of these biomarkers leading to biologically interpretable results with respect to their classification abilities, e.g., for diagnostic purposes. In contrast, in this paper we assumed *structural biomarkers*, derived from gene regulatory networks inferred from gene expression data, and used these to conduct a classification of disease states. From our numerical analysis we found that gene expression biomarkers and eigenvalue-based features perform similarly, although, the gene expression biomarkers perform slightly better.

This result is interesting because it demonstrates, first, a biomarker does not need to be a gene but it can be an abstract property of a biological system, e.g., eigenvalue-based network measures, as in our case. In principle this idea is not new. However, what is new is that we demonstrate this explicitly by giving an example for structural biomarkers. As such, we provide practical evidence to this argument which usually is only discussed argumentatively instead of numerically. Second, the way our structural biomarkers are defined does no longer allow to say, e.g., ‘gene A and gene B’ are able to distinguish the biological conditions under consideration. Instead, our features, respectively biomarkers, correspond to features of the *system* and are as such gene independent, but reflect their collective properties, as captured by the inferred gene regulatory networks. Hence, our approach represents a practical realization of *systems medicine*.

For a future analysis it would be interesting to use protein expression data rather than gene expression data to repeat a similar analysis. Such an analysis would allow to gain insights into the robustness of our results with respect to a change of the molecular level, as provided by protein interactions. Specifically, it would help to understand if pure [49] or mixed interaction types, as represented by gene regulatory networks, are better suited for constructing structural biomarkers.

Overall, our results provide promising evidence that *none-gene biomarkers* can be a beneficial means to classify disease states from gene expression data for diagnostic purposes.

### Appendix

For completeness, in the Tables 4, 5, 6, 7, 8, 9, 10 we show the same results as in Table 3 but for the individual data sets, as listed in Table 1.

**Table 4:** ndV-values for the structural biomarkers (eigenvalue and entropy-based measures) for prostate cancer networks and the corresponding subgroups (benign/cancer) for [50].

	absolute			relative		
	all	benign	cancer	all	benign	cancer
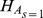	0	0	0	0	0	0
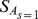	0	0	0	0	0	0
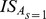	0	0	0	0	0	0
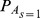	9	8	0	0.39	0.36	0
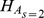	0	0	0	0	0	0
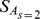	0	0	0	0	0	0
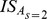	0	0	0	0	0	0
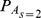	7	7	0	0.3	0.32	0
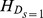	0	0	0	0	0	0
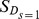	0	0	0	0	0	0
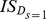	0	0	0	0	0	0
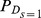	2	2	0	0.09	0.09	0
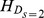	0	0	0	0	0	0
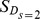	0	0	0	0	0	0
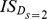	0	0	0	0	0	0
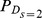	0	0	0	0	0	0
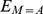	0	0	0	0	0	0
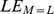	0	0	0	0	0	0
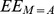	0	0	0	0	0	0
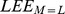	0	0	0	0	0	0
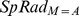	0	0	0	0	0	0

**Table 5:** ndV-values for the structural biomarkers (eigenvalue and entropy-based measures) for prostate cancer networks and the corresponding subgroups (benign/cancer) for [51].

	absolute			relative		
	all	benign	cancer	all	benign	cancer
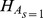	0	0	0	0	0	0
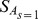	0	0	0	0	0	0
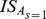	0	0	0	0	0	0
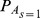	29	15	14	0.94	0.94	0.93
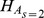	0	0	0	0	0	0
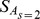	0	0	0	0	0	0
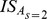	0	0	0	0	0	0
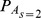	26	12	14	0.84	0.75	0.93
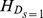	0	0	0	0	0	0
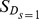	0	0	0	0	0	0
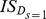	0	0	0	0	0	0
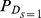	2	0	0	0.06	0	0
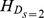	0	0	0	0	0	0
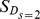	0	0	0	0	0	0
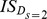	0	0	0	0	0	0
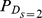	0	0	0	0	0	0
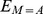	0	0	0	0	0	0
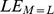	0	0	0	0	0	0
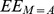	0	0	0	0	0	0
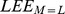	0	0	0	0	0	0
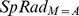	0	0	0	0	0	0

**Table 6:** ndV-values for the structural biomarkers (eigenvalue and entropy-based measures) for prostate cancer networks and the corresponding subgroups (benign/cancer) for [52].

	absolute			relative		
	all	benign	cancer	all	benign	cancer
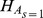	0	0	0	0	0	0
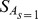	0	0	0	0	0	0
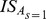	0	0	0	0	0	0
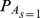	12	8	3	0.18	0.21	0.08
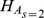	0	0	0	0	0	0
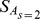	0	0	0	0	0	0
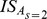	0	0	0	0	0	0
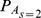	8	3	4	0.12	0.08	0.11
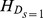	0	0	0	0	0	0
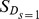	0	0	0	0	0	0
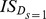	0	0	0	0	0	0
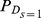	2	0	2	0.03	0	0.05
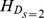	0	0	0	0	0	0
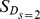	0	0	0	0	0	0
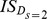	2	0	2	0.03	0	0.05
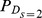	0	0	0	0	0	0
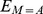	0	0	0	0	0	0
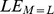	0	0	0	0	0	0
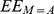	0	0	0	0	0	0
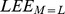	0	0	0	0	0	0
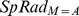	0	0	0	0	0	0

**Table 7:** ndV-values for the structural biomarkers (eigenvalue and entropy-based measures) for prostate cancer networks and the corresponding subgroups (benign/cancer) for [53].

	absolute			relative		
	all	benign	cancer	all	benign	cancer
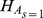	0	0	0	0	0	0
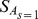	0	0	0	0	0	0
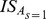	0	0	0	0	0	0
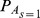	19	10	9	0.49	0.34	0.09
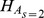	0	0	0	0	0	0
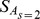	0	0	0	0	0	0
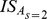	0	0	0	0	0	0
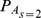	12	5	7	0.31	0.17	0.07
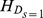	0	0	0	0	0	0
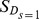	0	0	0	0	0	0
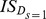	0	0	0	0	0	0
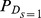	3	2	0	0.08	0.07	0
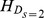	0	0	0	0	0	0
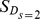	0	0	0	0	0	0
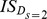	0	0	0	0	0	0
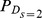	0	0	0	0	0	0
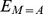	0	0	0	0	0	0
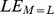	0	0	0	0	0	0
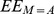	0	0	0	0	0	0
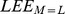	0	0	0	0	0	0
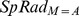	0	0	0	0	0	0

**Table 8:** ndV-values for the structural biomarkers (eigenvalue and entropy-based measures) for prostate cancer networks and the corresponding subgroups (benign/cancer) for [54].

	absolute			relative		
	all	benign	cancer	all	benign	cancer
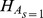	0	0	0	0	0	0
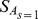	0	0	0	0	0	0
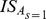	0	0	0	0	0	0
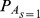	13	12	0	0.42	0.40	0
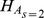	0	0	0	0	0	0
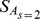	0	0	0	0	0	0
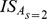	0	0	0	0	0	0
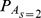	10	9	0	0.32	0.30	0
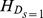	0	0	0	0	0	0
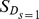	0	0	0	0	0	0
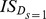	0	0	0	0	0	0
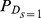	15	2	0	0.48	0.07	0
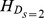	0	0	0	0	0	0
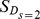	0	0	0	0	0	0
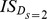	0	0	0	0	0	0
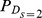	0	0	0	0	0	0
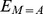	0	0	0	0	0	0
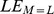	0	0	0	0	0	0
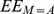	0	0	0	0	0	0
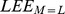	0	0	0	0	0	0
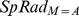	0	0	0	0	0	0

**Table 9:** ndV-values for the structural biomarkers (eigenvalue and entropy-based measures) for prostate cancer networks and the corresponding subgroups (benign/cancer) for [55].

	absolute			relative		
	all	benign	cancer	all	benign	cancer
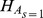	0	0	0	0	0	0
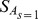	0	0	0	0	0	0
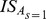	0	0	0	0	0	0
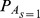	19	11	8	0.46	0.58	0.36
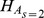	0	0	0	0	0	0
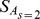	0	0	0	0	0	0
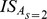	0	0	0	0	0	0
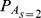	15	8	7	0.37	0.42	0.32
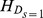	0	0	0	0	0	0
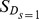	0	0	0	0	0	0
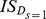	0	0	0	0	0	0
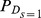	0	0	0	0	0	0
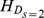	0	0	0	0	0	0
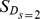	0	0	0	0	0	0
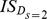	0	0	0	0	0	0
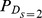	0	0	0	0	0	0
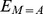	0	0	0	0	0	0
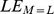	0	0	0	0	0	0
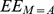	0	0	0	0	0	0
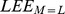	0	0	0	0	0	0
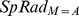	0	0	0	0	0	0

**Table 10:** ndV-values for the structural biomarkers (eigenvalue and entropy-based measures) for prostate cancer networks and the corresponding subgroups (benign/cancer) for [56].

	absolute			relative		
	all	benign	cancer	all	benign	cancer
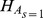	0	0	0	0	0	0
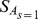	0	0	0	0	0	0
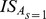	0	0	0	0	0	0
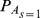	26	13	13	0.81	1	0.68
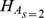	0	0	0	0	0	0
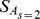	0	0	0	0	0	0
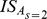	0	0	0	0	0	0
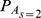	23	13	10	0.72	1	0.53
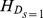	0	0	0	0	0	0
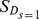	0	0	0	0	0	0
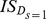	0	0	0	0	0	0
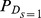	3	0	2	0.09	0	0.11
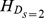	0	0	0	0	0	0
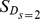	0	0	0	0	0	0
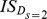	0	0	0	0	0	0
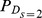	0	0	0	0	0	0
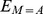	0	0	0	0	0	0
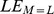	0	0	0	0	0	0
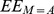	0	0	0	0	0	0
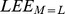	0	0	0	0	0	0
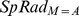	0	0	0	0	0	0

## Supporting Information

File S1 R data file containing descriptor values.
(ZIP)

File S2 Excel file containg the descriptor values by using eigenvalue-based measures.
(CSV)

File S3 Excel file containg the descriptor values by non-eigenvalue-based measures.
(CSV)
